# Case Report: Pulmonary and Liver Sarcoidosis Suspected of Metastasis

**DOI:** 10.12688/f1000research.13787.2

**Published:** 2018-06-04

**Authors:** Behnam Jafari, Gholamabas Sabz, Elahe Masnavi, Roghaye Panahi, Saeid Jokar, Amrollah Roozbehi, Sajad Hasanzadeh

**Affiliations:** 1 Student Research Committee, Yasuj University of Medical Sciences, Yasuj, Iran; 2 Department of Obstetrics and Gynecology, Yasuj University of Medical Sciences, Yasuj, Iran; 3 Department of Otolaryngology, Yasuj University of Medical Sciences, Yasuj, Iran; 4 Department of Dental Medicine, Yasuj University of Medical Sciences, Yasuj, Iran; 5 Department of Internal Medicine, Yasuj University of Medical Sciences, Yasuj, Iran; 6 Cellular and Molecular Research Center, Yasuj University of Medical Sciences, Yasuj, Iran

**Keywords:** Sarcoidosis, liver, lung, hilar lymphadenopathy

## Abstract

**Introduction**: Sarcoidosis is a granulomatous disease with unknown cause that can vary from an asymptomatic condition. Almost half of the patients with sarcoidosis have no symptoms. In this article, we describe a sarcoidosis patient with lung and liver engagement; it may be confused with metastasis.

**Case report**: A 39-year-old man, with known as hypothyroidism who had come to the emergency ward with dyspnea and coughing after exposure to detergents in a closed environment. The patient smoked for 10 years (3 pack/year). No other findings were found in clinical examinations except for wheezing in the right lung. The patient's chest radiography was shown a mass. For further investigation, spiral CT scan was performed. Large lymph nodes on the right side of the trachea, measuring about 23 mm and a mass of 70 × 77 mm in the vicinity of the right lung hilum and a hypodense nodule in the posterior part of the liver with malignancy suspicious were reported. After several biopsy results was shown chronic granulomatous inflammation, the most important differential diagnosis is tuberculosis (TB) and sarcoidosis. Sputum smear, culture, and PCR were performed for tuberculosis. Also, the level of angiotensin-converting enzyme (ACE) was measured for sarcoidosis. the results ruled out TB and shown a higher level of ACE (ACE = 88 IU/L).After diagnosis treatment started with prednisolone. Now, the patient is in the follow- up.

**Conclusion**: In hilar lymphadenopathy of lung sarcoidosis is the importance differential diagnosis that should be considered.

## Introduction

Sarcoidosis is a granulomatous disease with unknown cause that can vary from an asymptomatic condition to being life-threatening ^1^. Almost half of the patients with sarcoidosis have no symptoms. In some cases, with the help of chest radiography findings looking for other pathologies, it is diagnosed. Since the lungs are often involved, patients usually come to the clinic with lung complaints (such as shortness of breath, cough) ^2, 3^. Some of the clinical manifestations of sarcoidosis have a poor prognosis, including treatment-resistant lung sarcoidosis (pulmonary fibrosis, pulmonary hypertension), cardiac sarcoidosis, neurosarcoidosis, and multiple organ sarcoidosis. These clinical manifestations are often not diagnosed until the end stage of the disease, and their response to treatment is low. The incidence of severe sarcoidosis is the most common cause of death ^4^. On time diagnosis, treatment and follow-up of patients reduces mortality. In this article, we describe a sarcoidosis patient with lung and liver involvement, which may be misdiagnosed as cancer metastasis.

## Case report

**Figure 1.**
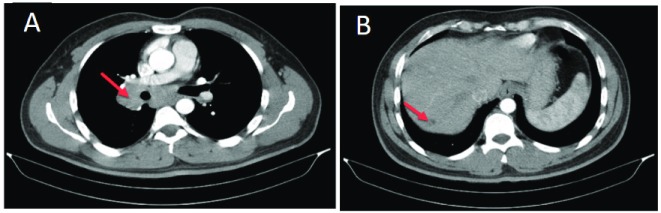
CT scan in-patient with sarcoidosis-( **A**) Pulmonary lymphadenopathy and ( **B**) granulomatous lesion in Liver involvement.

**Figure 2.**
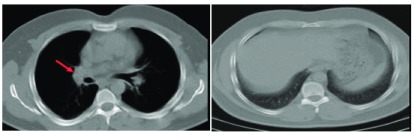
CT scan of patient 6 months after treatment showing decreased size of pulmonary lymphadenopathy and ( **A**) and improvement of hepatic lesions ( **B**).

**Figure 3.**
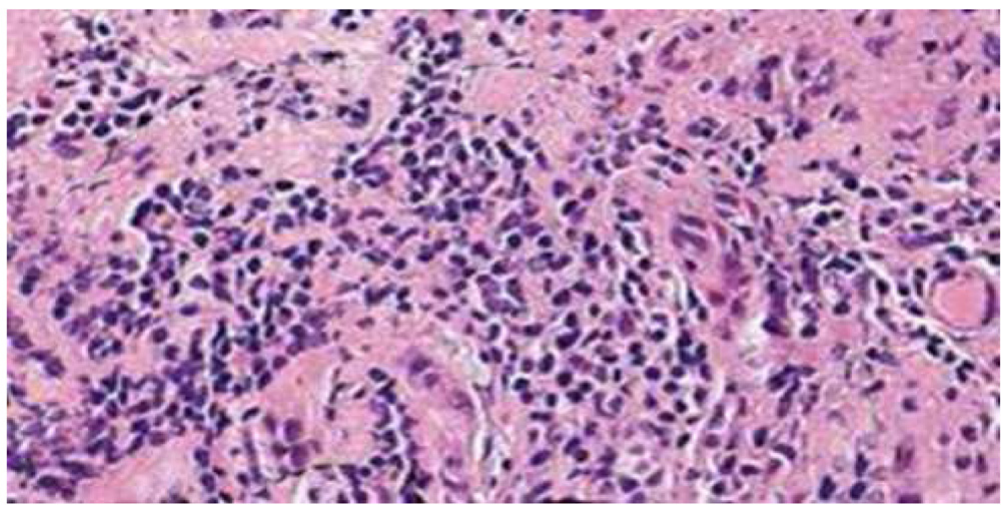
Pathology of Biopsy reveals infiltration of inflammatory cells.

A 39-year-old man, taxi driver, known to have hypothyroidism (being treated with levothyroxine) presented to the emergency ward with dyspnea and coughing after exposure to detergents in a closed environment. The patient was smoker (3 pack/year). The only in clinical examinations except for wheezing in the right side of chest. The patient's chest radiography identified a mass. For further investigation, a spiral computerized tomography (CT) scan was performed. Lymph nodes were enlarged on the right side of the trachea, measuring about 23 mm with a mass of 70 × 77 mm, in the vicinity of the right lung hilum. A hypodense nodule in the posterior part of the liver, suspected to be malignant, was also reported ( Figure 1). The lesions were suspected to be metastatic tumors, therefore, a biopsy of the mass was performed via bronchoscopy. The biopsy results were reported as chronic inflammation and mucosal hyperplasia without malignancy ( Figure 3), which did not conform to the CT report. The CT has repeated again, and confirmed the previous CT report. A CT guided mass biopsy was performed for pathological evaluation. The result showed chronic granulomatous inflammation, the two most likely causes being tuberculosis (TB) and sarcoidosis. Sputum smear, culture, and PCR were performed to test for TB, and angiotensin-converting enzyme (ACE) levels were measured for sarcoidosis. The results ruled out TB and showed high levels of ACE (ACE = 88 IU/L (normal 8-53)). In report of bronchoscopy, Vocal cord, trachea was normal. Carina was edematous that lead to narrowing of lumen of right bronchus. Two months after the first visit, sarcoidosis was diagnosed and treatment started with prednisolone. Ophthalmology test for eye evaluation, echocardiography for cardiac evaluation and EMG/NCV (Electromyogram test and nerve conduction study) for evaluation of the nervous system were also performed to determine if there was any extra-pulmonary sarcoidosis, however, no lesions were found. After treatment by corticosteroid the symptoms of the patient subsided. Now the patient is on follow-up. Figure 2 show the CT scan of patient after treatment.

## Discussion

Sarcoidosis occurs mainly in people aged 20 – 40 years and more common in females ^5^. Diagnosis is based on medical history, granuloma manifestations in at least two different structures, staining and negative culture for acid-fast bacilli, a lack of occupational and internal exposure to toxins and absence of drug-related illnesses. It is difficult to determine the prevalence and incidence of sarcoidosis since the number of patients who have sarcoidosis but do not have symptoms is unknown. In countries where *Mycobacterium tuberculosis* is common, sarcoidosis may not be detected ^5– 7^. The first evaluation of patients suspected of sarcoidosis include; cell blood count, serum biochemistry including creatinine, calcium, liver enzymes, alkaline phosphates, urine analysis, serum protein electrophoresis, inflammatory markers, lactate dehydrogenase, level of enzyme (ACE), and Complete Pulmonary Function Tests that should be performed in patients with respiratory symptoms or abnormalities of the lung parenchyma. In all cases, *Mycobacterium* and fungal disease should be considered, as it has a similar history (chronic cough) and clinical image (pulmonary lymphadenopathy) ^8^. A few clinical case report has shown that sarcoidosis is less common in smokers ^9, 10^. Sarcoidosis has many clinical manifestations and affects all organs of the body. The lung is involved in at least 90% of sarcoidosis patients. Skin, eyes, liver and peripheral lymph nodes, with a frequency of 10 to 30% for other involved organs ^10^. Cardiac involvement occurs in 25% of cases, but only causes clinical problems in 5% of cases, although it may suddenly be fatal, so it is important to examine all patients for cardiac sarcoidosis ^11, 12^. All patients should also be screened for eye involvement as it can cause visual impairment ^13^. Sarcoidosis, like drugs, poisons, viral infections, and flukes induce liver dysfunction ^14, 15^. Up to 35% patients have abnormal liver function tests that are not related to the degree of disease ^16, 17^. Serum levels of ACE are increased in 60% of patients and have been shown to correlate with the level of disease activity. This test is non-invasive and is highly effective because the enzyme is produced by epithelial granuloma cells and its serum level reflecting the entire granulomatous activity in the body ^8, 18^. Fatigue was reported in over 50% of patients, which has a major effect on the quality of life. Pain was reported in 70% of patients. Arthralgia is the most common type of pain; a headache and chest pain also being reported ^7, 19^. Sarcoidosis treatment, such as rheumatologic diseases and vasculitis, is based on corticosteroid. In terms of treatment, Corticosteroids are a selective therapeutic drug, and methotrexate and hydroxychloroquine are often alternative drugs ^20– 23^. Our patient had no abnormal blood counts, liver test dysfunction, and non-specific inflammatory markers abnormalities, only an increased titer of ACE with addition liver symptoms that included itching, jaundice, fever and abdominal pain.

## Conclusion

In hilar lymphadenopathy of lung, sarcoidosis is an importance differential diagnosis that should be considered and prior to biopsy of lymph nodes and any invasive procedures, ACE enzyme levels should be measured.

## Data availability

The data referenced by this article are under copyright with the following copyright statement: Copyright: © 2018 Jafari B et al.

Data associated with the article are available under the terms of the Creative Commons Zero "No rights reserved" data waiver (CC0 1.0 Public domain dedication). 

No data is associated with this article.

## Consent

Written informed consent was obtained from the patient for the publication of the patient’s clinical details and accompanying images.
