# Supplementary material for: Case Report: Pulmonary and Liver Sarcoidosis Suspected of Metastasis
Source: F1000Res. 2018 Jun 4;7:288. Originally published 2018 Mar 7. [Version 2] doi: 10.12688/f1000research.13787.2 (PMC5989148; doi:10.12688/f1000research.13787.2)
Supplement: Peer review history

# Peer review: Referee response for version 2

**DOI:** 10.5256/f1000research.16442.r34654

**Published:** 2018-06-04

**Authors:** Seyed Masoom Masoompour

**Affiliations:** 1 Non-Communicable Diseases Research Center, Shiraz University of Medical Sciences, Shiraz, Iran

Considering the authors respond to major comments, I think the manuscript is now acceptable for indexing.

I have read this submission. I believe that I have an appropriate level of expertise to confirm that it is of an acceptable scientific standard.

# Peer review: Referee response for version 1

**DOI:** 10.5256/f1000research.14986.r33755

**Published:** 2018-05-15

**Authors:** Edward S. Chen

**Affiliations:** 1 Division of Pulmonary and Critical Care Medicine, Department of Medicine, Johns Hopkins University, Baltimore, MD, USA

The authors present a case of pulmonary sarcoidosis arising in a country where tuberculosis may be more common.

 There is an error in the Abstract where a sentence is repeated " **A 39-year-old man, known as hypothyroidism who had come to the emergency ward with dyspnea and coughing after exposure to detergents in a closed environment **A 39-year-old man, known as hypothyroidism who had come to the emergency ward with dyspnea and coughing after exposure to detergents in a closed environment."

 In the abstract, the authors should revise the sentence "A 39-year-old man, known as hypothyroidism..." this probably should be revised to "A 39-year-old man **with known **hypothyroidism..."

 On page 3, please revise the sentence "The incidence of severe sarcoidosis is the most common cause of death."

 On page 3, the authors should provide information from the bronchoscopy procedure report as to whether there was airway narrowing visualized during the bronchoscopy. This is of particular interest since the patient's main radiographic abnormality is enlarged thoracic lymph nodes without any notable lung infiltrates (stage I chest x-ray).

 On Page 4, please consider revising "It is difficult to determine the prevalence and incidence of sarcoidosis without clinical symptoms." Perhaps rephrase to clarify that "It is difficult to determine the prevalence and incidence of sarcoidosis since the number of patients who have sarcoidosis but do not have symptoms is unknown."

I have read this submission. I believe that I have an appropriate level of expertise to confirm that it is of an acceptable scientific standard.

# Peer review: Referee response for version 1

**DOI:** 10.5256/f1000research.14986.r31753

**Published:** 2018-03-19

**Authors:** Seyed Masoom Masoompour

**Affiliations:** 1 Non-Communicable Diseases Research Center, Shiraz University of Medical Sciences, Shiraz, Iran

This case study presents a man with cough and dyspnea following exposure with irritant agent. His chest imaging showed a right hilar mass. Although this is not an unusual presentation of sarcoidosis but confirms that not all mass is malignant.

 My comments:

1. What do you mean by "The incidence of severe sarcoidosis is the most common cause of death"? As Swigris et al. had published, did you mean "The underlying cause of death in most patients with sarcoidosis was the disease itself."
2. Would you please clarify and rephrase this sentence: "The only in clinical examinations except for wheezing in the right side of chest" 
3. The authors please add the bronchoscopic findings; was there any mass or narrowing there. 
4. Please add the coronal view of chest CT; considering chest physical finding, wheeze, the reader would like to see if there is any narrowing in right main and/or intermediate bronchus.
5. Please add the unit of measurement of ACE level.
6. Please add the histologic slide of biopsy to the manuscript.

I have read this submission. I believe that I have an appropriate level of expertise to confirm that it is of an acceptable scientific standard, however I have significant reservations, as outlined above.
